# Neutrophil Extracellular Traps and Macrophage Extracellular Traps Predict Postoperative Recurrence in Resectable Nonfunctional Pancreatic Neuroendocrine Tumors

**DOI:** 10.3389/fimmu.2021.577517

**Published:** 2021-05-18

**Authors:** Shuai-Shuai Xu, Hao Li, Tian-Jiao Li, Shuo Li, Huan-Yu Xia, Jiang Long, Chun-Tao Wu, Wen-Quan Wang, Wu-Hu Zhang, He-Li Gao, Xuan Han, Long-Yun Ye, Xuan Lin, Hua-Xiang Xu, Xian-Jun Yu, Liang Liu

**Affiliations:** ^1^ Department of Pancreatic Surgery, Fudan University Shanghai Cancer Center, Shanghai, China; ^2^ Department of Oncology, Shanghai Medical College, Fudan University, Shanghai, China; ^3^ Shanghai Pancreatic Cancer Institute, Shanghai, China; ^4^ Pancreatic Cancer Institute, Fudan University, Shanghai, China

**Keywords:** prognosis, extracellular traps, macrophages, neutrophils, nonfunctional pancreatic neuroendocrine tumor, nomogram

## Abstract

**Background:**

Extracellular traps (ETs) and tumor-infiltrating immune cells can contribute to disease progression. The clinical significance of tumor-infiltrating neutrophils and macrophages and related extracellular traps in pancreatic neuroendocrine tumors (pNETs) has not been fully elucidated. This study aimed to explore the prognostic value of tumor infiltration and ET formation by neutrophils and macrophages in pNETs.

**Methods:**

A total of 135 patients with radical resection of nonfunctional pNETs were analyzed retrospectively. Immunohistochemistry and immunofluorescence were utilized to stain tumor tissue sections. The recurrence-free survival (RFS) of subgroups determined by Kaplan-Meier analysis was compared with the log-rank test. Univariate and multivariate Cox regression analyses were used to identify independent prognostic factors. A nomogram was established to predict 3-year RFS.

**Results:**

Patients with high tumor-infiltrating neutrophils or macrophages or positive expression of neutrophils ETs or macrophage ETs displayed worse RFS (all p<0.05). Moreover, univariate and multivariate Cox regression analyses showed that neutrophil and macrophage infiltration and ETs were independent prognostic factors for RFS (all p<0.05). A combined parameter including WHO grade, TNM stage, tumor-infiltrating neutrophils and macrophages, and neutrophil and macrophage ETs had the highest C-index (0.866) and lowest Akaike information criteria (326.557). The calibration plot of nomogram composed of the combined parameter exhibited excellent prognostic values for 3-year RFS.

**Conclusions:**

Infiltration and ETs by neutrophils and macrophages can be used as biological indicators of patient prognosis, suggesting the treatment potential for targeting those in nonfunctional pNETs.

## Introduction

Pancreatic neuroendocrine tumors (pNETs) are the second most common type of malignant tumor in the pancreas, and the morbidity shows an upward trend (1, 2). It is classified into functional and nonfunctional types with obvious heterogeneity, and most tumors are nonfunctional (3). At present, surgery remains the only possible treatment to cure this disease. Although the tumor grade classification system of the World Health Organization (WHO) for pNETs is regarded as the best tool to predict clinical outcomes, treatments have different effects on patients with tumors classified in the same grade or treated with similar therapeutic strategies (4). Therefore, the treatment of pNETs needs more attention. It is necessary to explore more accurate predictive tools for precision treatments based on each patient’s conditions (5, 6).

Current clinical drugs that are used to treat nonfunctional pNETs mainly target tumor cells (7, 8). However, the tumor microenvironment in pNETs, including immune cells, stromal cells, and extracellular substances, has an important role in tumor progression (9, 10). The roles of immune cells in tumor progression have been reported in many tumors, and corresponding immunotherapies have been recommended in clinical treatment guidelines or are being evaluated in clinical trials for certain tumors (11). Notably, the death processes of immune cells, especially extracellular trap (ET) formation, also influence disease progression. ET formation is considered a unique cell death process and is characterized by the production of mesh structures composed of the DNA skeleton, histones, granular proteins, and cytoplasmic proteins by immune cells after stimulation (12). Some studies have further noted that the citrullination of histones in cells plays a key role in the formation of immune cell ETs. The formation of immune cell ETs can help eliminate pathogens, but this mode of cell death may also disrupt the body’s normal development and homeostasis. Among immune cells, neutrophils and macrophages are known to produce ETs (13, 14). The production of ETs by other immune cells is still being explored. Neutrophil ETs contribute to the progression of diseases, such as systemic lupus erythematosus, shock, pancreatic cancer, and gastric cancer (15). Macrophage ETs participate in acute kidney injury (14), pathological conditions characterized by excessive hypochlorous acid formation (16), and antibacterial immunity (17). Nevertheless, no study has examined the role and mechanism of macrophage ETs in tumors until now. In addition, the association between ET formation and the infiltration of neutrophils and macrophages in tumor tissue is not clear.

In recent years, an increasing number of studies have reported the roles of neutrophil and macrophage infiltration in tumor progression (18). They not only enhance antigen presentation and feedback to the acquired immune system to mobilize and activate the whole immune system to kill tumor cells but also interact directly with tumor cells to exert influences (19). Many studies have revealed that macrophages can stimulate tumor-related angiogenesis, promote tumor invasion, metastasize into the blood vessels, resist chemotherapy, and induce antitumor immunity (20). The clinical role of tumor-infiltrating macrophages in pNETs has been partially elucidated (21). Neutrophils are an important class of innate immune cells that participate in different stages of the carcinogenic process, including tumor initiation, growth, proliferation, invasion, and metastasis (22). Tumor-infiltrating neutrophils in pNETs need to be further investigated.

In nonfunctional pNETs, the death process of immune cells, especially the clinical role of ETs, needs to be further discussed. The association between ET formation and the infiltration of neutrophils and macrophages into tumor tissue needs to be further confirmed. Crucially, few studies have explored the combined effects of immune cell infiltration and death formation in the tumor microenvironment on tumorigenesis and tumor development. Therefore, we studied the relationships between the infiltration or ET formation of neutrophils or macrophages and postoperative prognosis in nonfunctional pNETs. In addition, we also developed a nomogram, which is a predictive prognostic tool, with a combined indicator that included tumor-infiltrating neutrophils and macrophages and their related specific death by ET formation.

## Materials and Methods

### Patients

The inclusion criteria were as follows: (1) patients who underwent R0 resection were diagnosed histopathologically as having nonfunctional pNETs; (2) all patients received therapy under standardized guidelines; (3) preoperative and postoperative patients were routinely assessed according to clinical manifestations and auxiliary examinations, including laboratory tumor markers and imaging examinations; and (4) all cases included complete clinical preoperative and postoperative data. The exclusion criteria were as follows: (1) patients received presurgical antitumoral chemotherapy or radiotherapy; (2) patients had distant metastasis or a history of other malignant tumors; (3) patients received total pancreatectomy; (4) patients had multiple tumors in the pancreas; and (5) patients had postoperative complications, such as severe pancreatic fistula.

A total of 135 patients were retrospectively enrolled from 2013 to 2019. WHO grade was classified in compliance with the Ki-67 labeling index of the WHO guidelines established in 2017, and tumor-node-metastasis (TNM) stage was assessed according to the eighth edition of the American Joint Committee on Cancer guidelines. Recurrence-free survival (RFS) was defined as the interval from the date of surgery to the date of tumor recurrence or the last follow-up. This study was reviewed and approved by the Human Research Ethics Committee of Fudan University Shanghai Cancer Center and conformed to the tenets of the World Medical Association Declaration of Helsinki.

### Histopathological Assessment

Surgically resected formalin-fixed and paraffin-embedded specimens were evaluated with immunohistochemistry and immunofluorescence techniques. For immunohistochemistry, the paraffin sections were dewaxed, hydrated, washed with phosphate buffered saline (PBS), and then heated in a Tris-EDTA bath for antigen retrieval. After PBS washing, 1% bovine serum albumin was used for nonspecific antigen blocking at room temperature, and sections were left at 4°C overnight with primary antibody. Fluorescently labeled secondary antibody was added and incubated against light after washing with PBS. Cell nuclei were counterstained blue with DAPI. Primary antibodies were rabbit anti-citrullinated histone H3 antibody (ab5103, 1:200, Abcam), mouse anti-CD68 antibody (ab955, 1:100, Abcam), and goat anti-myeloperoxidase antibody (AF3667, 15 µg/mL, R&D). Fluorescently labeled secondary antibodies included donkey anti-rabbit IgG H&L (Alexa Fluor^®^ 488) antibody (ab150073, 1:400, Abcam), donkey anti-mouse IgG H&L (Alexa Fluor^®^ 647) antibody (ab150107, 1:400, Abcam), and donkey anti-goat IgG H&L (Alexa Fluor^®^ 647) antibody (ab150131, 1:400, Abcam). Macrophage ETs were specifically identified by positive staining with the anti-CD68 antibody and anti-citrullinated histone H3 antibody. Considering that myeloperoxidase and citrullinated histone H3 are also located in macrophage ETs (23), we calculated neutrophil ETs by subtracting macrophage ETs from myeloperoxidase-positive ETs, which were specifically identified by positive staining with the anti-myeloperoxidase antibody and anti-citrullinated histone H3 antibody (24, 25). The immunohistochemical technique was described in our previous study (26). The immunostaining images in serial sections from each case were estimated under high-power fields (HPF, magnification, ×200) to detect the number of neutrophils (CD15, ab135377, 1:50, Abcam) or macrophages (CD68, ab955, 1:100, Abcam) in tumor tissue. In addition, immunohistochemical and immunofluorescence assessments were performed by two independent experienced pathologists. Macrophage ETs and neutrophil ETs were stained in the same tissue using successive sections. For evaluation of immunohistochemical and immunofluorescence staining, five representative hotspot images were selected to count under HPF, and then the mean value was regarded as the number of final cell counts and final ETs in the tumor of each patient.

### Statistical Analysis

Neutrophil ETs and macrophage ETs were divided by whether positive expression was found in tumor tissue, and the cutoff values for other continuous variables were identified by the median. Continuous variables that were nonnormally distributed in different paired subgroups were compared by the Wilcoxon rank-sum test. Correlations of categorical variables were analyzed by Pearson’s chi-squared test or Spearman correlation analysis, while correlations of nonnormally distributed continuous variables were analyzed by Spearman correlation analysis. Kaplan-Meier survival curves were used to display RFS data, and differences between groups were compared with the log-rank test. Univariate and multivariate Cox regression analyses were used to identify independent prognostic factors for RFS. The concordance index (C-index) and Akaike information criterion (AIC) were used to compare the consistency and accuracy of the predictive models. A nomogram was established to predict 3-year RFS, and the calibration plot of the actual risk probability and predicted risk probability determined by the nomogram was used to display the predictive value of the prognostic models. All tests were two-sided, and p<0.05 was considered statistically significant. Statistical analyses were performed using SPSS software (version 20, IBM, Armonk, NY, USA) and R software (version 3.6.3, R Core Team, Vienna, Austria).

## Results

### Clinicopathological Features of Patients

The clinical features of all the patients are described in **Table 1**. The median age was 51 years, and 60 (44.4%) patients were male. In addition, fewer than half of the patients had pancreatic head tumors (45.9%), nerve invasion (33.3%), or vessel invasion (36.3%). The majority of patients (77.8%) had TNM stage I or II disease, and most of the tumors (92.6%) were grade 1 or grade 2. At the last follow-up, 12 patients (8.9%) had died, and 43 patients (31.9%) had experienced recurrence. Moreover, the 1-year, 3-year, and 5-year recurrence rates were 13.3%, 30.1%, and 35.3%, respectively. The mean RFS time was 50.6 months [95% confidence interval (CI): 46.2-54.9 months].

**Table 1:** Clinical characteristics of patients.

Clinical characteristics	n=135
Age, (median, interquartile range), years old	51 (43, 61)
Sex, male/female	60/75
Tumor location, head/body or tail	62/73
Nerve invasion, no/yes	90/45
Vessel invasion, no/yes	86/49
WHO grade, 1/2/3	68/57/10
TNM stage, I/II/III	33/72/30
Death, no/yes	123/12
Recurrence, no/yes	92/43

World Health Organization (WHO) grade was classified based on the Ki-67 labeling index of WHO guidelines established in 2017, and tumor-node-metastasis (TNM) stage was assessed according to the eighth edition of the American Joint Committee on Cancer guidelines.

### Characteristics of Neutrophil and Macrophage Infiltration and ETs

**Figure 1:**
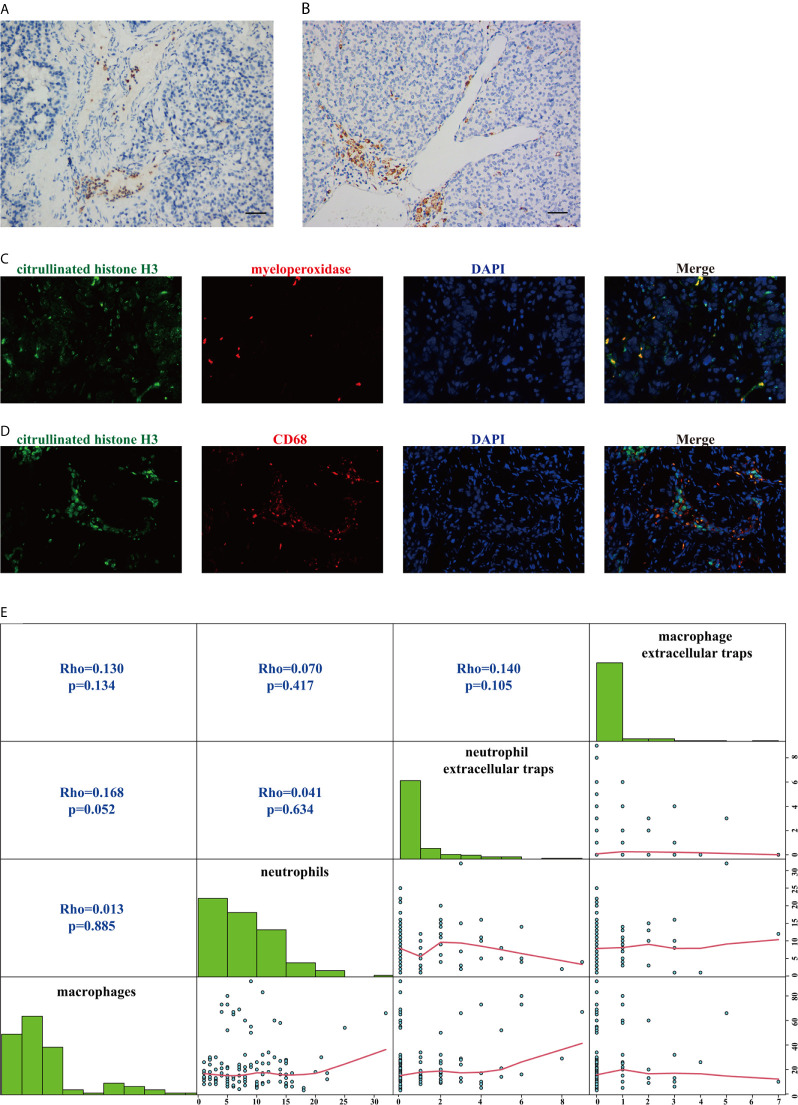
Staining patterns and the correlation of tumor-infiltrating neutrophils, tumor-infiltrating macrophages, neutrophil extracellular traps, and macrophage extracellular traps. **(A)** Representative immunohistochemical staining for tumor-infiltrating neutrophils (CD15). Scale bar, 50 μm. **(B)** Representative immunohistochemical staining for tumor-infiltrating macrophages (CD68). Scale bar, 50 μm. **(C)** Representative immunofluorescence staining for myeloperoxidase-positive extracellular traps (magnification, 400×). **(D)** Representative immunofluorescence staining for macrophage extracellular traps (magnification, 400×). **(E)** Matrix plots to assess the correlations among tumor-infiltrating neutrophils, tumor-infiltrating macrophages, neutrophil extracellular traps, and macrophage extracellular traps. The correlations of nonnormally distributed continuous variables were analyzed by Spearman correlation analysis.

As shown in **Figures 1A, B**, neutrophils and macrophages were stained in each section. Macrophages were more numerous than neutrophils in the tumor tissue (median, 17/HPF *vs* 8/HPF; p<0.001). When the median was used as the cutoff value, the infiltration of macrophages was not correlated with the clinical characteristics of the patients (**Table 2**, all p>0.05), but the infiltration of neutrophils was associated with WHO grade (**Table 2**, p=0.010). There was no significant correlation between the infiltration of neutrophils and that of macrophages (**Figure 1E**, p=0.885 and **Table 2**, p=0.102).

**Table 2:** P values for associations between neutrophil or macrophage infiltration or extracellular traps and clinicopathological factors.

Factors	Neutrophil infiltration	Macrophage infiltration	Neutrophil extracellular traps	Macrophage extracellular traps
Age	0.143	0.344	0.091	0.796
Sex	0.264	0.939	0.135	0.386
Tumor location	0.339	0.339	0.194	0.057
Nerve invasion	0.808	0.330	0.798	0.361
Vessel invasion	0.057	0.188	0.439	0.421
WHO grade	0.010	0.247	0.015	0.684
TNM stage	0.102	0.264	0.433	0.087
Neutrophil infiltration	–	0.102	0.401	0.143
Macrophage infiltration	0.102	–	0.229	0.143
Neutrophil extracellular traps	0.401	0.229	–	0.104
Macrophage extracellular traps	0.143	0.143	0.104	–

All factors used were categorical variables. The correlations of the categorical variables were analyzed by Pearson’s chi-squared test or Spearman correlation analysis.

The representative patterns of myeloperoxidase-positive ETs and macrophage ETs are shown in **Figures 1C, D**. The positive ET expression rates in neutrophils and macrophages were 34.8% (47/135) and 20% (27/135), respectively, and they were significantly different (p=0.001). Positive staining for macrophage ETs was independent of the clinical characteristics of the patients (**Table 2**, all p>0.05), but positive staining for neutrophil ETs was associated with WHO grade (**Table 2**, p=0.015). Positive staining expression for neutrophil ETs and that for macrophage ETs were not significantly correlated (**Figure 1E**, p=0.105 and **Table 2**, p=0.104). Additionally, neither positive staining for neutrophil ETs nor that for macrophage ETs showed a significant correlation with the infiltration of neutrophils or macrophages in tumor tissue (**Figure 1E** and **Table 2**, all p>0.05).

### Correlations Between Neutrophil and Macrophage Infiltration and ETs and the Survival of Patients

**Figure 2:**
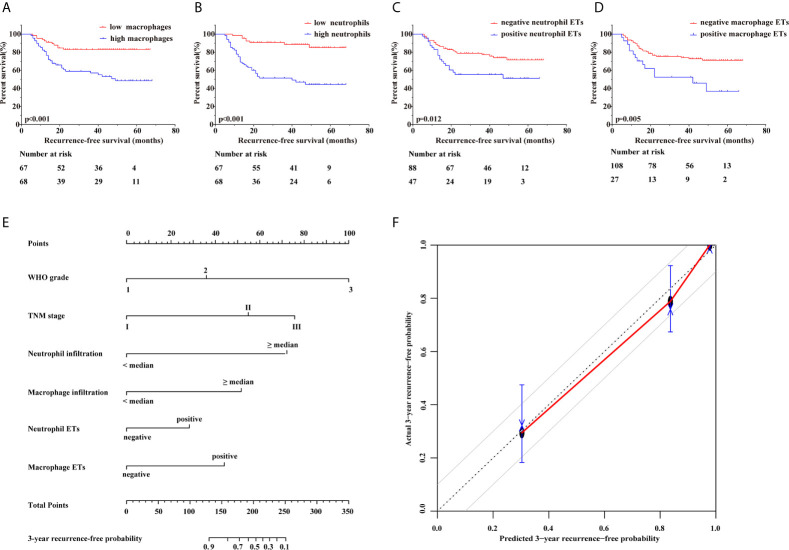
Correlations between neutrophil and macrophage infiltration and ETs and the recurrence outcome of patients. **(A)** Kaplan–Meier curves showed that high tumor-infiltrating macrophages predicted significantly shortened recurrence-free survival (p<0.001). **(B)** Kaplan–Meier curves showed that high tumor-infiltrating neutrophils predicted significantly shortened recurrence-free survival (p<0.001). **(C)** Kaplan–Meier curves showed that the positive presence of neutrophil extracellular traps predicted significantly shortened recurrence-free survival (p=0.012). **(D)** Kaplan–Meier curves showed that the positive presence of macrophage extracellular traps predicted significantly shortened recurrence-free survival (p=0.005). **(E)** A nomogram for 3-year recurrence-free predictive probability. **(F)** Calibration plot for 3-year recurrence-free survival. Calibration plots show the actual risk probability with the 95% confidence interval overpredicted risk probability, and the dashed line corresponds to the 10% margin of error.

Kaplan-Meier survival curves showed (**Figures 2A–D**) that high tumor-infiltrating neutrophil and macrophage numbers were associated with poorer RFS (both p<0.001) than low tumor-infiltrating neutrophil and macrophage numbers. Similar results were found for the presence of neutrophil ETs or macrophage ETs, which was associated with shortened RFS (p=0.012, p=0.005, respectively). Furthermore, univariate and multivariate Cox regression analyses (**Table 3**) showed that the number of tumor-infiltrating neutrophils or macrophages, the presence of neutrophil ETs or macrophage ETs, WHO grade, and TNM stage were independent prognostic factors for RFS (all p<0.05).

**Table 3:** Cox regression analyses for recurrence-free survival with clinicopathological factors.

Factors	Univariate Cox regression			Multivariate Cox regression		
	HR	95% CI	P value	HR	95% CI	P value
Age (< 51/≥ 51), years old	0.707	0.387-1.291	0.259			
Sex (male/female)	1.210	0.656-2.230	0.541			
Tumor location (head/body or tail)	1.205	0.657-2.209	0.547			
Nerve invasion (no/yes)	1.321	0.717-2.435	0.373			
Vessel invasion (no/yes)	0.806	0.426-1.525	0.507			
WHO grade (1/2)	4.721	2.203-10.116	<0.001	2.451	1.041-5.769	0.040
WHO grade (1/3)	15.749	5.951-41.678	<0.001	12.136	3.983-36.975	<0.001
TNM stage (I/II)	3.975	1.196-13.206	0.024	3.862	1.020-14.627	0.047
TNM stage (I/III)	8.349	2.430-28.687	0.001	6.660	1.564-28.356	0.010
Neutrophils (< 8/≥ 8), per HPF	5.739	2.658-12.391	<0.001	5.987	2.652-13.516	<0.001
Macrophages (< 17/≥ 17), per HPF	3.344	1.684-6.639	0.001	3.675	1.777-7.601	<0.001
Neutrophil extracellular traps (negative/positive)	2.118	1.163-3.857	0.014	2.035	1.043-3.970	0.037
Macrophage extracellular traps (negative/positive)	2.433	1.283-4.615	0.006	3.017	1.493-6.096	0.002

CI, Confidence interval; HR, Hazard ratio; All factors used were categorical variables. WHO grades 2 and 3 were compared with WHO grade 1. TNM stages Ⅱ and Ⅲ were compared with TNM stage Ⅰ.

### Neutrophil and Macrophage Infiltration and ETs as Supplemental Prognostic Factors Used to Establish a Nomogram

Based on the aforementioned conclusions, neutrophil and macrophage infiltration and ETs should be regarded as predictive prognostic parameters. To assess the consistency and accuracy of predictive models (**Table 4**), we chose independent prognostic factors for RFS to establish prognostic models and found that a combined parameter including WHO grade, TNM stage, tumor-infiltrating neutrophils and macrophages, neutrophil ETs, and macrophage ETs had the highest C-index (0.866) and lowest AIC (326.557), surpassing WHO grade, TNM stage, and a parameter incorporating both. Therefore, WHO grade combined with TNM stage, tumor-infiltrating neutrophils and macrophages, neutrophil ETs, and macrophage ETs were selected for inclusion in a nomogram. Considering that the cumulative incidence of 3-year recurrence after resection reaches up to 26.5% (27) and that 2-year RFS is an important recurrence risk score group analysis time point (28), we selected 3-year survival as the observed long-term survival. Nomograms showed remarkably accurate clinical usefulness to predict recurrence and survival outcomes, superior to current predictive prognosis models (29). A nomogram was established to predict the 3-year recurrence-free probability (**Figure 2E**). In addition, the calibration plot of the nomogram (**Figure 2F**) exhibited excellent prognostic values and centralized mainly in the 10% margin of error for 3-year predictive recurrence-free probability.

**Table 4:** Comparisons of prognostic models for recurrence-free survival.

Prognostic models	C-index	AIC
WHO grade	0.738	369.164
TNM stage	0.676	337.852
Neutrophil infiltration	0.700	374.527
Macrophage infiltration	0.635	387.147
Neutrophil extracellular traps	0.595	395.140
Macrophage extracellular traps	0.577	394.457
WHO grade+ TNM stage	0.787	367.077
WHO grade+ TNM stage+ neutrophil infiltration+ macrophage infiltration+ neutrophil extracellular traps+ macrophage extracellular traps	0.866	326.557

C-index, concordance index; AIC, Akaike information criterion.

## Discussion

Current prognostic models for nonfunctional pNETs mainly depend on WHO grade and TNM stage. However, innate immune cells participate in numerous processes, including the infiltration, invasion, and metastasis of tumor cells (30). Of note, not only the infiltration status but also ETs, a typical product produced during the death process in neutrophils and macrophages, affect the survival outcomes of patients with pNETs. We analyzed the immune signatures of neutrophils and macrophages in pNETs, highlighting that integrated immune indicators produced better recurrence prediction models than individual markers.

Our immunofluorescence experiments showed that there were relatively fewer neutrophil ETs and macrophage ETs than tumor-infiltrating neutrophils and macrophages in tumor tissue. Nevertheless, neutrophil ETs and macrophage ETs were independent risk factors for tumor prognosis, and positive ET staining was associated with reduced RFS. We further speculated that neutrophil and macrophage ETs have significant effects on tumor recurrence in nonfunctional pNETs. The formation of neutrophil ETs is inseparable from the multicellular interactions among neutrophils, tumor cells, platelets, and endothelial cells, which are related to disease progression, metastasis, and venous thrombosis (31, 32). Cancer cells promote neutrophil ET formation and are closely related to the inflammatory cytokines ICAM-1, VCAM-1, E-selectin, IL1, IL6, CXCL1, and C3a receptors (33, 34). On the other hand, the formation of neutrophil ETs can regulate mitochondrial function and Toll-like receptors of tumor cells to promote the growth and metastasis of tumor cells (35–37) and induce the proliferation of dormant tumor cells (24). It prevents contact between tumor cells and CD8-positive T cells and natural killer cells to inhibit antitumor immunity (38) and can adjust tumor-associated fibroblasts in the tumor microenvironment (39). Additionally, it mediates resistance to chemotherapy drugs and immunosuppressive point therapy (40) and is associated with the hypercoagulable state of blood vessels, which leads to cancer-related thrombosis (41). In addition, it promotes adhesion, proliferation, and metastasis of cancer cells under a postoperative stress state (42). Therefore, neutrophil ETs and tumor cells form a mutually reinforcing loop to expedite tumor progression. Macrophage ETs show features similar to those of neutrophil ETs. They can be produced *via* protein arginine deiminase 2 and driven by TNF-α from adipose cells (43). However, implicated cellular pathways and functions have received less attention. Our study revealed that the prognosis of patients with positive macrophage ETs was worse than that of patients with negative expression. The related molecular mechanisms of macrophage ET formation and the interaction between macrophage ETs and tumor cells deserve further exploration in nonfunctional pNETs. The four sets of parameters, including tumor-infiltrating neutrophil numbers, tumor-infiltrating macrophage numbers, neutrophil ETs, and macrophage ETs, had no significant correlations but showed significant differences, suggesting that the tumor infiltration status and the formation of ETs may be regulated by different mechanisms in distinct immune cells.

Neutrophils can be attracted by tumor cells through CXCR2 ligands and, in return, can also affect tumor cell invasion through tyrosine receptors (44). Neutrophils secrete matrix metalloproteinase-9 and other substances to promote tumorigenesis and induce angiogenesis (45). They weaken the antitumor effect of CD8-positive T cells *via* metalloproteinase activation (46) and recruit macrophages and regulatory T cells to facilitate tumor progression and drug resistance (47). In our study, we found that low tumor-infiltrating neutrophil numbers were associated with prolonged RFS. It is suggested that we need to consider further therapy to regulate neutrophils in pNETs. Our results also showed that high tumor-infiltrating macrophage numbers were associated with relatively early recurrence. Macrophage numbers can also be related to prognosis and used as an important supplement for WHO grade, which agrees with the findings of a study by Cai L et al. (21), which implied that macrophages accelerated tumor progression in pNETs. Macrophage activation can be regulated by cancer cells associated with lysosome-associated membrane protein type 2A (48). Macrophages also provide feedback to tumor cells (49). They stimulate neovascularization by activating the p38/MAPKAP kinase 2 axis (50) and synergize with other cells in the tumor microenvironment, such as fibroblasts and T cells, to enhance tumor invasion and metastasis (51, 52). The infiltration of macrophages, especially that of tumor-associated M2 macrophages, has demonstrated a close connection with chemotherapy resistance in many tumors (53). Targeting macrophages in pNETs could be seen as a new clinical strategy.

There are still some limitations to our research. First, this was a retrospective study of nonfunctional pNET patients with low morbidity. Second, other immune cells, immune molecules, and immune cell death processes may also influence patient prognosis. Finally, the mechanism underlying the interaction among neutrophils, macrophages, and immune cell ETs in pNETs needs further explanation.

## Conclusions

Recent immunotherapy schemes and drugs, such as CTLA-4, PD-1, and PD-L1 inhibitors that target immune checkpoints, have shown promising therapeutic effects in many tumors (54). However, a related immunotherapy strategy for pNETs has not been identified. Herein, we proposed associations between prognosis and neutrophil and macrophage infiltration and ETs in nonfunctional pNETs, suggesting the potential of immunotherapy strategies that regulate tumor-infiltrating neutrophils and macrophages, neutrophil ETs, and macrophage ETs. Our results provide a new indicator composed of WHO grade, TNM stage, and innate immune parameters, which can be utilized to predict patient prognosis and deserves to be explored in clinical application with chemotherapy and immunotherapy in nonfunctional pNETs. Notably, this study revealed that macrophage ETs also participated in tumor progression more than in anti-infection immunity and suggested the possibility that innate immune cells in tumor tissue support tumor progression *via* their infiltration and death.

## Data Availability Statement

The original contributions presented in the study are included in the article/supplementary material. Further inquiries can be directed to the corresponding authors.

## Ethics Statement

The studies involving human participants were reviewed and approved by the Human Research Ethics Committee of Fudan University Shanghai Cancer Center. The patients/participants provided their written informed consent to participate in this study.

## Author Contributions

S-SX, HL, T-JL, SL, H-YX, JL, W-HZ, W-QW, H-LG, and XH contributed to sample preparation and carried out the experiment. S-SX, HL, T-JL, and SL performed the research and discussed the data. S-SX, HL, T-JL, SL, H-YX, L-YY, XL, and C-TW conducted all statistical analyses and contributed to the implementation of the research and interpretation of data. X-HX, LL, and X-JY revised the manuscript and provided valuable suggestions for this study. All authors contributed to the article and approved the submitted version.

## Funding

This work was supported by grants from the National Science Foundation for Distinguished Young Scholars of China (81625016), the National Natural Science Foundation of China (81872366, 81871941, 81827807, 81802675, 81701630, and 81702341), the Outstanding Academic Leader Program of the “Technological Innovation Action Plan” of Shanghai Science and Technology Commission (18XD1401200), the Scientific Innovation Project of Shanghai Education Committee (2019-01-07-00-07-E00057), the Natural Science Foundation of Shanghai (19ZR1410800), the Clinical and Scientific Innovation Project of Shanghai Hospital Development Center (SHDC12018109), and the Young Talented Specialist Training Program of Shanghai.

## Conflict of Interest

The authors declare that the research was conducted in the absence of any commercial or financial relationships that could be construed as a potential conflict of interest.

The reviewer GS declared a shared affiliation with all of the authors, to the handling editor at time of review.
